# Aggregation of alpha-synuclein disrupts mitochondrial metabolism and induce mitophagy via cardiolipin externalization

**DOI:** 10.1038/s41419-023-06251-8

**Published:** 2023-11-10

**Authors:** Olivier Lurette, Rebeca Martín-Jiménez, Mehtab Khan, Razan Sheta, Stéphanie Jean, Mia Schofield, Maxime Teixeira, Raquel Rodriguez-Aller, Isabelle Perron, Abid Oueslati, Etienne Hebert-Chatelain

**Affiliations:** 1 Canada Research Chair in Mitochondrial Signaling and Physiopathology, Moncton, NB Canada; 2 Department of Biology, University of Moncton, Moncton, NB Canada; 3 CHU de Québec Research Center, Axe Neurosciences, Quebec City, QC Canada; 4 Department of Molecular Medecine, Université Laval, Quebec City, QC Canada

**Keywords:** Mechanisms of disease, Pathogenesis

## Abstract

Accumulation of α-synuclein aggregates in the substantia nigra pars compacta is central in the pathophysiology of Parkinson’s disease, leading to the degeneration of dopaminergic neurons and the manifestation of motor symptoms. Although several PD models mimic the pathological accumulation of α-synuclein after overexpression, they do not allow for controlling and monitoring its aggregation. We recently generated a new optogenetic tool by which we can spatiotemporally control the aggregation of α-synuclein using a light-induced protein aggregation system. Using this innovative tool, we aimed to characterize the impact of α-synuclein clustering on mitochondria, whose activity is crucial to maintain neuronal survival. We observed that aggregates of α-synuclein transiently and dynamically interact with mitochondria, leading to mitochondrial depolarization, lower ATP production, mitochondrial fragmentation and degradation via cardiolipin externalization-dependent mitophagy. Aggregation of α-synuclein also leads to lower mitochondrial content in human dopaminergic neurons and in mouse midbrain. Interestingly, overexpression of α-synuclein alone did not induce mitochondrial degradation. This work is among the first to clearly discriminate between the impact of α-synuclein overexpression and aggregation on mitochondria. This study thus represents a new framework to characterize the role of mitochondria in PD.

## Introduction

Parkinson’s disease (PD) is characterized by the specific degeneration of dopaminergic (DA) neurons in the substantia nigra pars compacta (SNc), leading to a dramatic depletion of cerebral dopamine and the apparition of motor symptoms, including tremor, bradykinesia, and muscle rigidity [1, 2]. PD is also characterized by the accumulation of inclusions called Lewy bodies (LBs) in the cytosol of neurons [3, 4]. LBs distribution in the brain coincides with neuronal loss in vulnerable populations, notably the SNc [1–3]. Neuropathological observations have revealed a correlation between the presence of cortical LBs and cognitive impairments in patients suffering from PD [5, 6]. Furthermore, deposition of LBs at dendritic neurites is considered a key pathological event at early stages of PD and other related disorders [7, 8]. LBs tend to accumulate several neuronal proteins but are mainly constituted of the presynaptic protein alpha-synuclein (α-syn) [4]. Evidence also suggests that α-syn fibrillization and aggregation are the major events in LBs formation [3, 4]. Moreover, mutations (A30P, E46K, A53T, H50Q, G51D) of the gene coding for α-syn (*SNCA*) which precipitate α-syn aggregation are all associated with familial forms of PD [3, 4, 9]. Collectively, these observations highlight the role of LBs in PD pathogenesis, as well as in disease progression. The mechanisms by which LBs induce neuronal loss remain however elusive.

Brain activity critically depends on the high energetic support provided by mitochondria. These organelles are the main converters of cellular energy sources into ATP thanks to the electron transport system and the oxidative phosphorylation process (OXPHOS). ATP is used in the brain for many different specific processes, including neurotransmitter release and synaptic plasticity [10]. Mitochondria are very dynamic cellular elements, forming complex networks within the cytoplasm of the cells, constantly modified by processes of fission and fusion, which determine their functional properties [11, 12]. In brain cells, mitochondrial transport to different subcellular compartments is an important mechanism allowing for the presence of the organelles at the sites where high-energy supply or other functions are required [13, 14].

Converging evidence from neuropathological, genetic and experimental studies support the important role of mitochondrial dysfunction in PD pathogenesis. The first evidence of mitochondrial dysfunction in PD pathogenesis was reported after the accidental induction of PD-like symptoms in young drug abusers exposed to 1-methyl-4-phenyl-1,2,3,4-tetrahydropyridine (MPTP), a potent inhibitor of the complex I of the electron transport chain (ETC). Later, post-mortem analysis from PD-diseased brain samples revealed a significant decrease of complex I enzymatic activity [15, 16] and increased abundance of mitochondrial DNA mutations [17, 18], reflecting profound mitochondrial dysfunction associated with the disease. Additionally, genetic evidence shows that the majority of the PD-associated genes, such as *PINK1* and *PRKN*, either encode mitochondrial proteins or are associated with mitochondrial functions, and their mutations result in a deep disruption of mitochondrial functions [17]. Finally, experimental models of PD reported that treatments with mitochondrial toxins, i.e. MPTP and rotenone, induce a selective degeneration of the DA neurons in rodent brains [19, 20].

The role of LBs formation on induction of mitochondrial dysfunctions is however not yet fully elucidated. Increasing lines of evidence indicate that monomeric α-syn interacts with mitochondria and modulates fusion and fission events between individual mitochondria, transport and degradation of the organelles [21]. For instance, ultrastructural studies have reported that monomeric α-syn is present in the outer and inner mitochondrial membranes, as well as in the mitochondrial matrix [21]. By interacting with key mitochondrial components, such as the voltage dependent anion-selective channel (VDAC) or the translocase of outer membrane (TOM), α-syn induces mitochondrial dysfunction [22, 23]. Alpha-synuclein is also observed in mitochondria-associated endoplasmic reticulum (ER) membranes (MAMs) which mediate Ca^2+^ transfer between the two organelles [23]. However, how α-syn oligomers, fibrils and aggregates specifically interact with mitochondria remains scantly understood. Overexpression of PD-linked mutant *SNCA* prone to aggregation leads to disrupted association between ER and mitochondria, altered Ca^2+^ homeostasis, enhanced mitochondrial fragmentation and depolarization, inhibition of complex I of the electron transport system and neuronal loss [23, 24]. These findings illustrate that α-syn overexpression can lead to alterations of mitochondrial homeostasis. However, the overexpression of any protein can lead to cellular stress, and overexpressed α-syn could affect mitochondria independently of its aggregation. Moreover, many studies using overexpression of α-syn without observing the presence of α-syn aggregates noticed mitochondrial defects [25–31]. Considering that most cellular and animal models of PD use overexpression of α-syn but do not control the aggregation of α-syn, the exact mechanisms by which α-syn aggregation and LBs formation affect mitochondria remain elusive.

We recently generated a new optogenetic tool enabling to control spatiotemporally α-syn aggregation [32]. This tool is using the capacity of the *Arabidopsis thaliana*’s cryptochrome protein 2 (CRYolig2) to form rapid protein clusters when exposed to blue light [33, 34], allowing to generate a light-induced protein aggregation (LIPA) tool. The photoreceptor CRYolig 2 was fused to mCherry and α-syn (LIPA-α-syn) enabling to control the aggregation of α-syn. Two control LIPA constructs were also generated: CRYolig 2 fused to mCherry (named LIPA-Empty) and CRYolig 2 fused to mCherry and a nonaggregatable form of α-syn, missing the non-amyloid-β component (NAC) region NAC essential for α-syn aggregation [35] (named LIPA-α-syn^ΔNAC^). Altogether, these constructs allow to examine and discriminate between the impact of α-syn overexpression and aggregation as well as between α-syn monomers and aggregates. Importantly, LIPA constructs mimic several authentic features of LBs both in vitro and in vivo [32]. In the present work, we took advantage of the LIPA system to investigate how α-syn overexpression and aggregation impact on mitochondria, using different cell lines, human dopaminergic neurons and mouse SNc. We observed that α-syn aggregation blunted mitochondrial membrane potential and ATP levels, resulting in fragmentation and degradation of mitochondria via cardiolipin externalization-dependent mitophagy, whereas overexpression of α-syn alone had no impact on mitochondrial content.

## Methods

### Cell culture, DNA transfection and stimulation of LIPA

HeLa, N2A and HEK cells were obtained from ATCC and were routinely tested for mycoplasma contamination. Cells were maintained in high glucose (4,5 g/L) Dulbecco’s modified Eagle’s medium (DMEM) supplemented with 2 mM glutamine, 1 mM pyruvate, 10% (v/v) of fetal bovine serum (FBS), 100 units/mL penicillin and 100 g/mL streptomycin. Cells were cultured at 37 °C in 5% CO_2_ and 95% humidity.

Human dopaminergic neurons were derived from human iPSCs kindly provided by Dr. Thomas Durcan (McGill University, Canada). Differentiation of iPSCs into dopaminergic neurons was performed as described [32].

HeLa and N2A cells were transfected with polyethylenimine (PolySciences, PA, USA). Human dopaminergic neurons were transfected using Lipofectamine 3000 (ThermoFisher Scientific, USA) following manufacturer’s instructions seven days post-differentiation. HEK cells stably expressing LIPA constructs were generated as described [32].

Mito-GFP, Drp1-EYFP and Drp1-K38E-EYFP were generously shared by Julien Prudent (University of Cambridge, UK.) Ateam constructs were kindly offered by Iromi Imaruma (Kyoto University, Japan). HA-ubiquitin-WT and HA-ubiquitin-KO were obtained from Addgene (plasmid #17608 and 17603, respectively). Plasmid encoding α-syn was provided by provided by Dr. Hilal Lashuel (EPFL, Switzerland).

Cells were stimulated with a blue LED light system (Prizmatix, Israel) at an intensity of 0.8 mW/mm^2^ without inducing phototoxicity [32, 33].

### Mice

Three-month-old C57/BL6 mice were obtained from Charles River laboratories and habituated for 7 days before handling. Mice were housed with 12 h light/dark cycle and ad libitum access to food and water.

AAVs encoding the LIPA constructs were stereotaxically injected in the SNc using the coordinates −3.08 mm posterior, −1.5 mm lateral, −4.25 mm ventral. To stimulate LIPA constructs in SNc, wireless optogenetic devices were implanted in SNc using the following coordinates −3.08 mm posterior, −1.5 mm lateral, −4.20 mm ventral, as described [32]. 15 days after surgery, in vivo light stimulation was performed every second day. Stimulation was performed for 1 h at the power of 1.76 mW/mm^2^ (measured at the tip of the optical fiber) and a pulse of 10 ms at 20 Hz, as we previously described [32].

### SDS-PAGE and western blotting

Cells were washed with cold PBS, collected by gentle scraping and centrifuged at 1500 *g* for 5 min (4 °C). After, pellets were resuspended in lysis buffer (150 mM NaCl, 20 mM Tris-HCl, pH 7.5, 1% Triton X-100) supplemented with 1% protease inhibitor cocktail, 2 mM sodium orthovanadate and 1 mM sodium fluoride. Samples were kept on ice during 20 min and then centrifugated at 12,500 *g* for 10 min (4 °C) to collect the TCL. The protein concentration was determined by the Bradford assay. Proteins were separated using 10% polyacrylamide gel containing 0.5% (v/v) of 2,2,2-trichloroethanol for staining loaded proteins. Voltage was initially set at 95 V during 15 min and then 155 V during 90 min. Proteins were blotted to PVDF membranes. Membranes were blocked for 1 h in TBS-T (50 mM Tris-Cl, pH 7.6; 150 mM NaCl, 0.1% Tween) containing 5% BSA or 5% skim milk and incubated with primary antibodies overnight at 4 °C. Protein immunodetection was performed using primary antibodies directed against aSyn (BD Transduction; #BD610787), mCherry (Abcam; #125096), actin (Abm; #G043), SDHA (Abcam; #ab14715), tubulin (Cell Signaling; #2144), BNIP3L (Cell Signaling; #12396), FUNDC1 (Cell Signaling; #49240) and PLSCR3 (ABclonal; #A12371). Membranes were then washed with TBS-T and incubated with appropriate peroxidase-conjugated anti-rabbit or anti-mouse secondary antibodies for 1 h at room temperature. After washing in TBS-T, immunoblots were visualized by chemiluminescence using the ChemiDoc Touch imaging system (Biorad, Irvine, CA, USA). The 2,2,2-tricholoroethanol added directly in SDS-PAGE gels interacts with tryptophan in loaded protein and induces UV light-induced fluorescence which can be visualized on a 300 nm transilluminator, as described [36]. The total UV light-induced fluorescence corresponds to the total protein load. All uncropped immunoblots are shown in supplemental file 1.

### Mitochondrial isolation

Cells were harvested, resuspended in mitochondrial isolation buffer (250 mM sucrose, 1 mM EDTA, 5 mM HEPES, pH 7.4) and lysed using a Potter-Elvehjem homogenizer. Cell debris and nuclei were removed by centrifugation at 500 g, and the supernatant was centrifuged at 12,500 g for 10 min (4 °C). The following pellet was resuspended in mitochondrial isolation buffer and a cycle of centrifugation at 1500 g and 12,500 g was repeated. The final pellet was considered as the mitochondria-enriched fraction, and was processed for further analyses.

### Cellular respiration

Oxygen consumption rates were measured with 1 × 10^6^ cells ml^− 1^ at 37 °C in 2 ml chambers of an Oroboros O2k oxygraphy, as described [37–39]. Three different states of endogenous respiration with intact cells were measured: (i) basal respiration representing the endogenous physiological coupled state, (ii) respiration with oligomycin (2 μg ml^− 1^) representing the non-coupled resting respiration, and (iii) maximal uncoupled respiration induced by FCCP (0.5 μM steps with 2 μM final concentration) providing a measure of the maximal capacity of ETS under conditions of physiological substrate supply in the intact cell.

### Immunofluorescence

Cells were seeded on 12 mm glass round coverslips coated with poly-L-lysine 0.05% (w/v). When appropriate, cells were fixed in 2% (w/v) paraformaldehyde, 5% sucrose (w/v) for 20 min at 37 °C. Cells were permeabilized with 0.15% triton X-100 in PBS for 15 min and then blocked in PBS containing 5% BSA for 45 min. Primary antibodies HA (Biolegend; #901501 or 902301), SDHA (Abcam; #ab14715), ATPB (Abcam; #ab14748), MAP2 (Cell Signaling Technology #4542 S), aSyn (BD Transduction; #BD610787) were incubated overnight at 4 °C. Coverslips were washed with PBS and then incubated with appropriate Alexa Fluor®-conjugated secondary antibody for 1 h at room temperature (RT) and protected from light. Coverslips were mounted on microscope slides with Vision™ PermaFluor™ Aqueous Mounting Medium (Richard-Allan Scientific, #TA-030-FM). Equal numbers of cells from the 4 quadrants of coverslips were imaged for each independent experiment. Cells were imaged using an Olympus FV3000 confocal microscope (Tokyo, Japan) with a 60X oil objective (UPLAN 60x oil, 1.35NA, Olympus) and appropriate excitation/emission parameters. Stacks separated by 0.2 μm along the z axis were acquired.

For SNc immunofluorescence, animals were sacrificed, and brains were removed after transcardial perfusion with 0.9% NaCl, followed by 4% PFA-PBS. After overnight fixation in 4% PFA, brains were placed in 4% agar, and cut in sagittal sections (100 μm thick), with a vibratome (Leica, Germany) and stored at 4 °C. Slices were washed 3 times (10 min) with PBS, incubated 1 h at RT in blocking buffer (3% BSA, 0.1% Triton X100-PBS), and then incubated overnight at 4 °C with primary antibody prepared in blocking buffer. The slices were then washed 3 times (10 min) with PBS and incubated 2 h at RT, with appropriate Alexa Fluor-conjugated secondary antibodies in 0.1% Triton X100-PBS. After, slices were washed 3 times (10 min) with 0.1% Triton X100- PBS, incubated in Hoechst 33342 and finally washed 2 times (10 min) with PBS. For microscopy imaging, slices were mounted in Vision™ PermaFluor™ Aqueous Mounting Medium. Slices were imaged using an Olympus FV3000 confocal microscope (Tokyo, Japan) with a 20× objective and appropriate excitation/emission parameters. One image of 400 μm × 400 μm in mCherry-positive SNc was acquired for each slice. Three slices from three independent mice in each group were analyzed. Total fluorescence of ATPB and Hoechst was measured with the Fiji software. ATPB fluorescence was then normalized by the Hoechst fluorescence.

#### Post-acquisition analyses

For assessing the total mitochondrial area (as a proxy for the total mitochondrial content) in cells, images were first compiled by “max projection”. Region of interests (ROIs) covering 40 × 40 μm for HEK cells and human dopaminergic neurons, 25 × 25 μm for HeLa cells, 45 × 45 μm for N2A cells were selected from max projection images and followed by manual thresholding, as previously described [40]. The total mitochondrial area in ROIs were obtained using the Analyze particles plugin in Fiji with a minimum area of 0.2 μm^2^. At least 10 cells were analyzed for each independent experiment.

Colocalization analysis was performed from max projection images using the appropriate plug-in in the Fiji software (NIH, USA). To examine colocalization between aggregates and mitochondria, the minimum pixel value of mCherry was increased to exclude pixels not labeling visible LIPA aggregates prior to analyses.

### Live microscopy

For assessing the mitochondrial membrane potential, live cells were incubated with 150 nM of MitoTracker™ Deep Red or 250 nM MitoTracker™ Red for 30 min and directly visualized with appropriate parameters. As of control, mock cells were pre-treated with 1 μm of FCCP during 5 min before staining with MitoTracker™ Deep Red.

For assessing oxidative stress, live cells were incubated with 25 µM of CM-H_2_DCFDA for 30 min and directly visualized with appropriate parameters. As of control, mock cells were pre-treated with 5 mM of H_2_O_2_ during 30 min before staining with CM-H_2_DCFDA.

For Ateam assays, cells expressing Ateam or mitoAteam were analyzed 24 h post-transfection, with excitation at 435 nm and emission at 485–520 nm for CFP and 530–600 nm for YFP. Fluorescence of CFP and YFP was acquired at T_0_ on a minimum of 8 cells for each independent experiment. Cells were then treated with 2-deoxyglucose (1 mM) and KCN (1 mM), and CFP and YFP fluorescence was acquired after 20 min (T_20_) on the same cells imaged at T_0_. The YFP/CFP ratio was finally measured from max projection images in a randomly selected ROI of 25 μm^2^ within peripheral regions of cells using Fiji. Data are reported as ΔATeam = T_20_-T_0_.

For live imaging, cells were seeded on 18 mm glass round coverslips coated with poly-L-lysine 0.05% (w/v). Before experiments, coverslips were transferred into Chamlide™ chambers and Okolab stage top incubator. One stack of 2 μm was imaged every ≈2 s during 5 min for HEK cells and 7.5 min for HeLa cells. The number of single contacts and the duration of these contacts were then manually followed. Individual contact between LIPA aggregates and mitochondria was noted when one or more pixels of a single LIPA aggregate overlapped with one or more pixels of one mitochondrion during at least two timeframes.

### Cardiolipin externalization

The relative cardiolipin amount on the surface of the outer mitochondrial membrane was evaluated as described previously [41] with minor modifications. First, cells were labeled with 312.5 nM Mitotracker™ Deep Red for 30 min at 37 °C. Subsequently, cells were harvested, and mitochondria were isolated as described above. Mitochondria were resuspended in Annexin V Binding Buffer (Biolegend) and incubated with FITC-labeled Annexin V (Biolegend) to detect anionic phospholipids through flow cytometry. The FITC fluorescence from gated far red fluorescent mitochondria events was determined to evaluate the binding of Annexin V to mitochondria. As positive control, cells were treated with 2 μM CCCP for 1 hour.

### RNAi

Cells were transduced with siRNAs 24 h before transfection with LIPA constructs. Cells were treated with 50 nM of scrambled siRNA (named siControl, ThermoFischer Scientific; #4390843), siBNIP3L (Sigma-Aldrich; #EHU033701), siFUNDC1 (Sigma-Aldrich; #EHU138091) or siPLSCR3 (Qiagen; #1027416). All siRNAs were transduced using the MISSION® siRNA Transfection Reagent (Sigma-Aldrich; #S1452) according to the manufacturer’s protocol. Cells were then stimulated under blue light 24 h post-transfection of LIPA constructs and 48 h post-transfection of siRNA.

### Statistical analyses

Data are presented as mean ± s.e.m. Statistical analyses were performed using GraphPad Prism 9. Data were assessed for normality and were analyzed using Student *t*-test, one-way or two-way ANOVA followed by Tukey post-hoc test, as appropriate. The exact sample size (i.e., independent biological replicates) for each group and condition corresponds to the number of data point in panels. Data points with different letters are statistically different (*p* < 0.05). For instance, a data point with the letter *a* is statistically different (*p* < 0.05) from data points with the letters *b* or *bc*, whereas it is not statistically different (*p* > 0.05) from data points with the letters *a* or *ab*.

## Results

### LIPA-induced α-syn aggregates dynamically interact with mitochondria

**Fig. 1:**
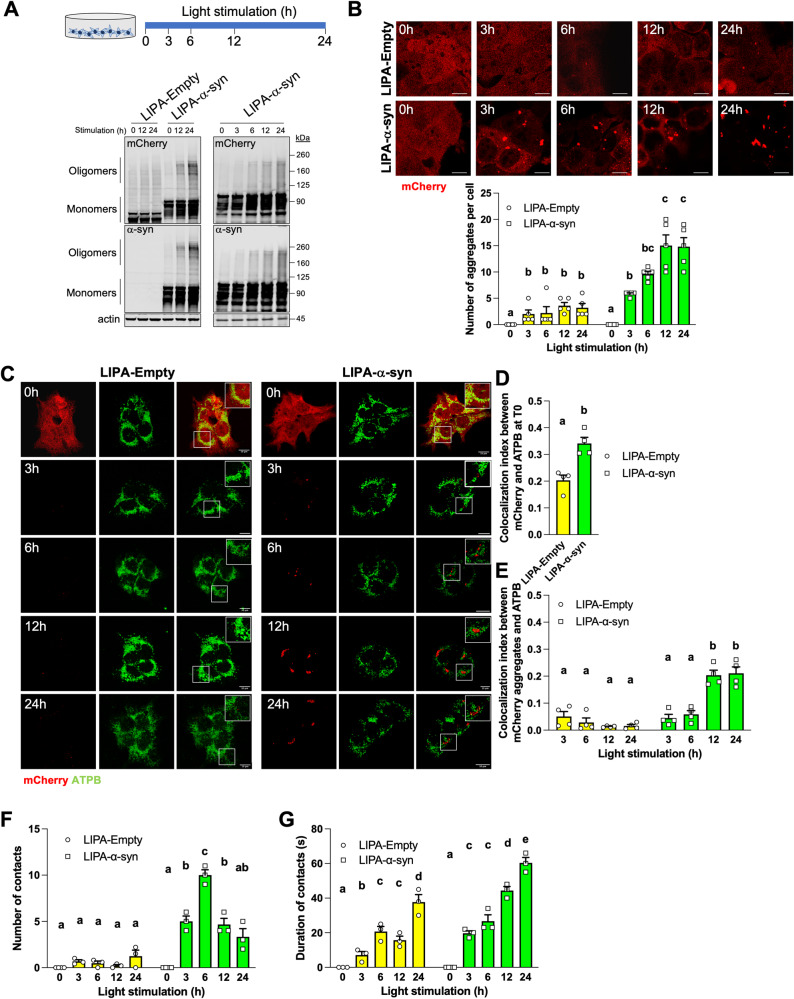
LIPA-induced α-syn aggregates transiently interact with mitochondria. **A** Schematic representation of the experimental paradigm used in Fig. 1. Representative immunoblotting (*n* = 3) of mCherry, α-syn and actin in HEK cells stably expressing LIPA-Empty and LIPA-α-syn at different time points during light stimulation, showing the time-course generation of high molecular LIPA-α-syn aggregates. **B** Representative micrographs and quantification of the number of aggregates in mCherry-positive HEK cells (*n* = 5) stably expressing LIPA constructs and stimulated as shown in (**A**). **C** Representative micrographs showing mCherry and the mitochondrial protein ATPB in HEK cells stably expressing the indicated LIPA constructs and stimulated for different time points. Note that pixel values were modified at T > 0 h to show only aggregates. **D**, **E** Pearson’s colocalization index between mCherry or aggregates and ATPB in unstimulated (**D**) and stimulated HEK (**E**) cells stably expressing the indicated LIPA constructs (*n* = 4). **F**, **G** Quantification of the number (**F**) and the duration (**G**) of individual contacts between aggregates and mitochondria during live imaging of HEK cells stably expressing LIPA constructs. See also Video 1-10. Scale bar: 10 μm. Data are mean ± s.e.m., and were analyzed by two-way ANOVA followed by Tukey post-hoc tests in (**B**, **E**, **F**, **G**) and by Student *t*-test in (**D**). Data points with different letters are statistically different (*p* < 0.05).

To visualize and monitor α-syn aggregates, we overexpressed and stimulated LIPA constructs in different cell lines. First, HEK cells stably expressing LIPA-Empty and LIPA-α-syn constructs (named hereafter HEK^LIPA-Empty^ and HEK^LIPA-α-syn^) were stimulated for different times (Fig. 1A). Immunoblotting of mCherry and α-syn showed a time-dependent increase in oligomers in HEK^LIPA-α-syn^ (Fig. 1A). The mCherry oligomers were also more abundant in HEK^LIPA-α-syn^ than in HEK^LIPA-Empty^ (Fig. 1A). Imaging of mCherry showed a similar time-dependent increase of aggregates in both stimulated HEK^LIPA-Empty^ and HEK^LIPA-α-syn^, with more aggregates in HEK^LIPA-α-syn^ than in HEK^LIPA-Empty^ (Fig. 1B).

LIPA-Empty and LIPA-α-syn constructs were also transiently expressed in HeLa and N2A cells. Stimulation with blue light increased the number of aggregates with both constructs in the two cell lines (Fig. S1A, B). Similar to stable HEK cells, HeLa expressing LIPA-Empty had less aggregates than cells expressing LIPA-α-syn after 24 h of light stimulation (Fig. S1A), whereas there was only a non-significant increase of aggregate numbers in N2A expressing LIPA-α-syn as compared to N2A expressing LIPA-Empty (Fig. S1B). Thus, the LIPA system allows to generate in a time-dependent manner aggregates of mCherry or aggregates of α-syn fused to mCherry.

In general, LIPA-α-syn produced more aggregates than LIPA-Empty (Figs. 1A, B and S1A, B), suggesting that the clustering capacity of the LIPA system is modulated by the aggregation capacity of α-syn. To further test this, we transiently expressed LIPA-α-syn^ΔNAC^ in HeLa and N2A cells. As observed previously [32], no aggregates were observed after light stimulation in these cells (Fig. S1A, B), indicating that the non-aggregatable α-syn^ΔNAC^ completely blocked the clustering capacity of the LIPA system. These results demonstrate that the aggregation capacity of α-syn is crucial to generate aggregates via the LIPA system.

We then explored whether LIPA constructs would interact with mitochondria before and after light stimulation. LIPA constructs appeared uniformly distributed in the cytosol of unstimulated cells although the colocalization between mCherry and the mitochondrial protein ATPB was higher in HEK^LIPA-α-syn^ than in HEK^LIPA-Empty^ (Fig. 1C, D). Colocalization between ATPB and the aggregates was also low in stimulated HEK^LIPA-Empty^ and HEK^LIPA-α-syn^, although the indexes slightly increased after 12 h and 24 h of light stimulation in HEK^LIPA-α-syn^ (Fig. 1C–E). Likewise, mCherry or LIPA aggregates poorly colocalized with ATPB before and after light stimulation, respectively, in HeLa and N2A cells (Fig. S1C, D).

Mitochondria are dynamic organelles constantly transported throughout the cytoplasm. Thus, colocalization might not be sufficient to describe the interaction between aggregates and the organelles. We performed live imaging of HEK^LIPA-Empty^ and HEK^LIPA-α-syn^ co-expressing mitochondria-targeted GFP (mitoGFP) to further examine this interaction (Videos 1–10). Surprisingly, videos showed that the aggregates physically interact with mitochondria in a kiss-and-run manner in stimulated HEK^LIPA-Empty^ and HEK^LIPA-α-syn^. Similar dynamic contacts were observed in live HeLa cells transiently co-expressing mitoGFP and the same LIPA constructs (Videos S1–S6). The number of unique contacts between the aggregates and mitochondria increased over time in HEK^LIPA-α-syn,^ but not in HEK^LIPA-Empty^, reaching a maximum after 6 h of light stimulation (Fig. 1F). Duration of these individual contacts increased over time in both HEK^LIPA-Empty^ and HEK^LIPA-α-syn^, whereas contacts usually lasted longer in HEK^LIPA-α-syn^ (Fig. 1G). Thus, albeit their overall different intracellular distribution, LIPA aggregates and mitochondria physically interact in a dynamic fashion.

### LIPA-induced α-syn aggregates decreases mitochondrial mass and alters OXPHOS

We observed important disruptions of mitochondrial morphology, trafficking and mass upon α-syn aggregation in live imaging experiments (Videos 1–10 and S1-6). These preliminary observations prompted us to examine more precisely and quantitatively the potential alterations of mitochondrial physiology induced by α-syn aggregation.

**Fig. 2:**
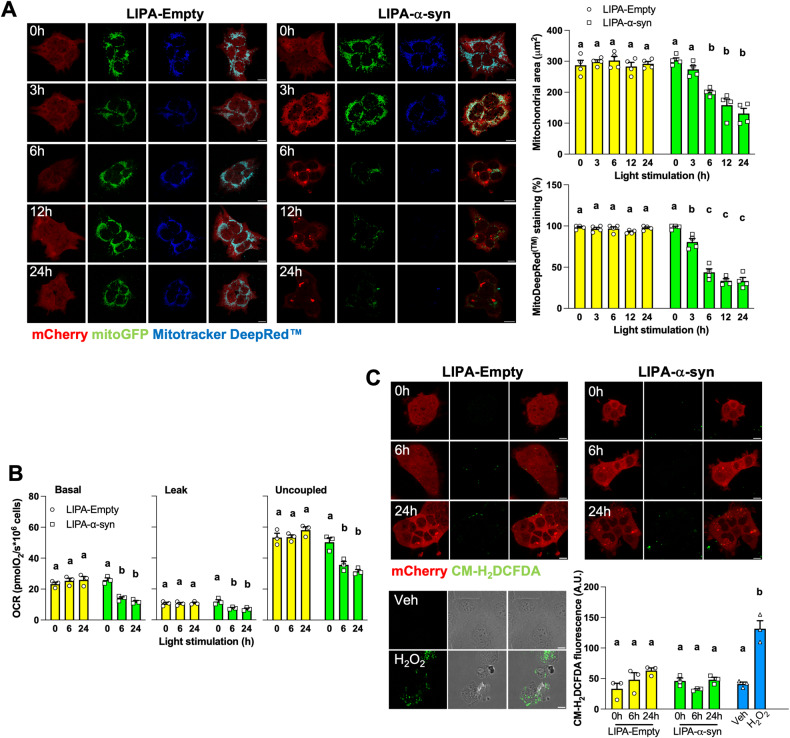
LIPA-induced α-syn aggregates decrease mitochondrial content and metabolism. **A** Representative micrographs and quantification of HEK co-expressing LIPA constructs and mitoGFP, stained with Mitotracker Deep Red™ and stimulated as indicated (*n* = 4). The area covered by mitoGFP was quantified as a proxy for the total mitochondrial content. **B** Oxygen consumption rates (OCR) of HEK cells expressing the LIPA constructs and stimulated as indicated (*n* = 3). **C** Representative micrographs and quantification of HEK cells expressing the LIPA constructs and of mock HEK cells treated with vehicle (veh) or H_2_O_2_ (5 mM during 30 min) stained with CM-H_2_DCFDA (*n* = 3). Scale bar: 10 μm. Data are mean ± s.e.m., and were analyzed by two-way ANOVA followed by Tukey post-hoc tests. Data points with different letters are statistically different (*p* < 0.05).

First, we analyzed mitochondrial content during stimulation of live HEK^LIPA-Empty^ and HEK^LIPA-α-syn^ expressing mitoGFP (Fig. 2A). The area covered by mitoGFP decreased over time in HEK^LIPA-α-syn^ but remained stable in HEK^LIPA-Empty^ (Fig. 2A). Similar decrease of mitochondrial content was also observed in HeLa cells transiently expressing LIPA-α-syn and stained with antibodies specific for ATPB or SDHA but not in HeLa cells expressing LIPA-Empty or LIPA-α-syn^ΔNAC^ (Fig. S2A, B).

Mitochondrial activity was then examined using various approaches. HEK^LIPA-Empty^ and HEK^LIPA-α-syn^ co-expressing mitoGFP were stained with Mitotracker™ Deep Red which labels mitochondria according to the mitochondrial membrane potential. Staining of Mitotracker™ Deep Red decreased overtime in HEK^LIPA-α-syn^ but not in HEK^LIPA-Empty^ (Fig. 2A). Moreover, staining with Mitotracker™ Deep Red of stimulated HeLa and N2A cells expressing LIPA-α-syn cells was similar to mock cells treated with the uncoupler FCCP, whereas no change was observed upon light stimulation in cells expressing LIPA-Empty or LIPA-α-syn^ΔNAC^ (Fig. S3A, B). Oxygen consumption also decreased during light stimulation of HEK^LIPA-α-syn^ but not in HEK^LIPA-Empty^ (Fig. 2B). ATP levels were then measured in live HeLa cells using the ATP FRET reporter Ateam [42]. This sensor has higher YFP/CFP ratio in the presence of high cytosolic ATP levels, whereas the YFP/CFP ratio decreases after treatment with inhibitors of ATP production [42]. HeLa cells co-expressing LIPA constructs and Ateam were treated with the glycolysis inhibitor 2-deoxyglucose and the cytochrome c oxidase (i.e., complex IV of the ETC) inhibitor KCN. Then, the difference of YFP/CFP ratios before and after treatment with these drugs (ΔYFP/CFP) was compared between unstimulated and stimulated cells. The Ateam ΔYFP/CFP was similar between unstimulated HeLa cells expressing the three LIPA constructs (Fig. S3C). Also, Ateam ΔYFP/CFP did not change upon stimulation of LIPA-Empty or LIPA-α-syn^ΔNAC^ (Fig. S3C). However, stimulation of HeLa cells expressing LIPA-α-syn decreased Ateam ΔYFP/CFP (Fig. S3C), indicating that the aggregation of α-syn decreases cytosolic ATP levels. We also examined ATP levels within mitochondria using the mitochondria-targeted mitoAteam. Similar to the results obtained with the untargeted Ateam, the mitoAteam ΔYFP/CFP decreased only upon stimulation of LIPA-α-syn (Fig. S3D). To examine whether LIPA-α-syn aggregates results in oxidative stress, we stained HEK^LIPA-Empty^ and HEK^LIPA-α-syn^ with CM-H_2_DCFDA, a general indicator for reactive oxygen species. Unexpectedly, no difference in CM-H_2_DCFDA staining was observed in HEK^LIPA-Empty^ and HEK^LIPA-α-syn^ during light stimulation (Fig. 2C). Altogether, our findings indicate that LIPA-α-syn aggregates decrease mitochondrial content and alter mitochondrial metabolism over time, whereas aggregates of LIPA-Empty lacking α-syn do not impact on mitochondria.

**Fig. 3:**
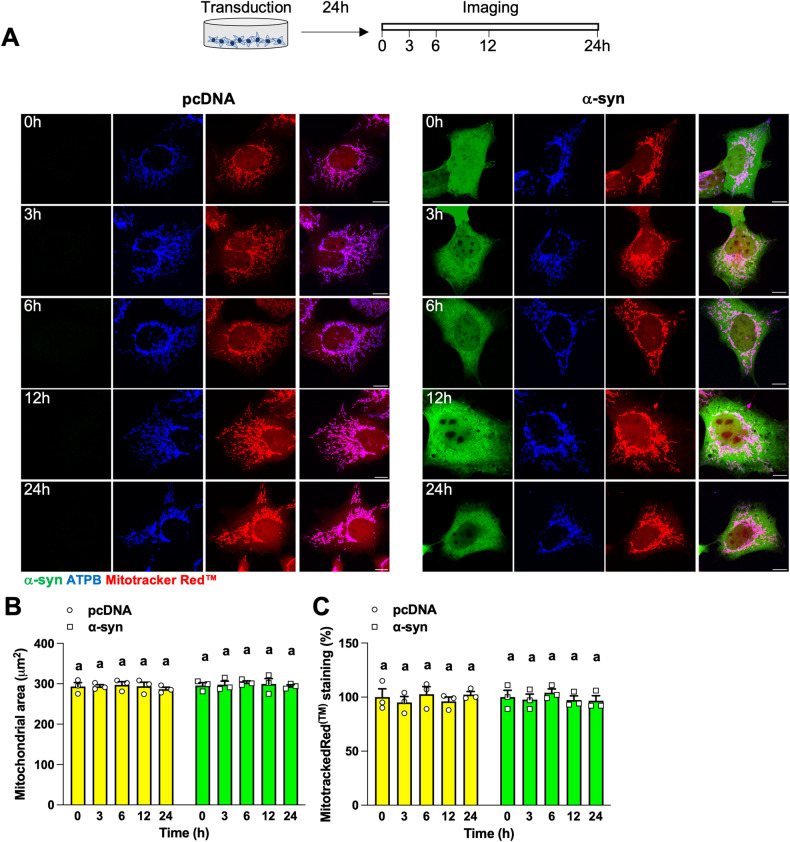
Overexpression of α-syn alone does not impair mitochondrial content and membrane potential. **A**–**C** HEK cells expressing pcDNA or α-syn were imaged at the indicated time points 24 h after transfection. Representative (*n* = 3) micrographs (**A**) and quantification of ATPB (**B**) and Mitotracker Red™ (**C**) staining (*n* = 3). The area covered by ATPB staining was quantified as a proxy for the total mitochondrial content. Scale bar: 10 μm. Data are mean ± s.e.m., and were analyzed by two-way ANOVA followed by Tukey post-hoc tests. Data points with different letters are statistically different (*p* < 0.05).

Mitochondrial content, Mitotracker™ Deep Red staining and cellular respiration were similar between unstimulated cells expressing LIPA-Empty and LIPA-α-syn (Figs. 2A, B, S2A, B, S3A, B) suggesting that the overexpression of α-syn is not sufficient to hamper mitochondria. To test this, we repeated the time-course analysis of mitochondrial content in non-stimulated HEK cells transiently expressing LIPA constructs or α-syn alone. As shown before [32], no aggregates visible by microscopy or immunoblotting were generated over time without light stimulation in HEK cells transiently expressing LIPA-Empty or LIPA-α-syn (S4A, B). Also, the area covered by ATPB staining did not change over time in these cells (Fig. S4A). Similarly, overexpression of α-syn alone was not accompanied with α-syn aggregates visible by IF and WB (Fig. 3A, S4B). Mitochondrial content and MitoTracker™ Red staining, another marker of mitochondrial membrane potential, were not different between cells expressing the control vector pcDNA or α-syn alone (Fig. 3B, C). These results suggest that the overexpression of α-syn is not sufficient to induce α-syn aggregates and impair mitochondria.

### LIPA-α-syn aggregates decrease mitochondrial content in human dopaminergic neurons

**Fig. 4:**
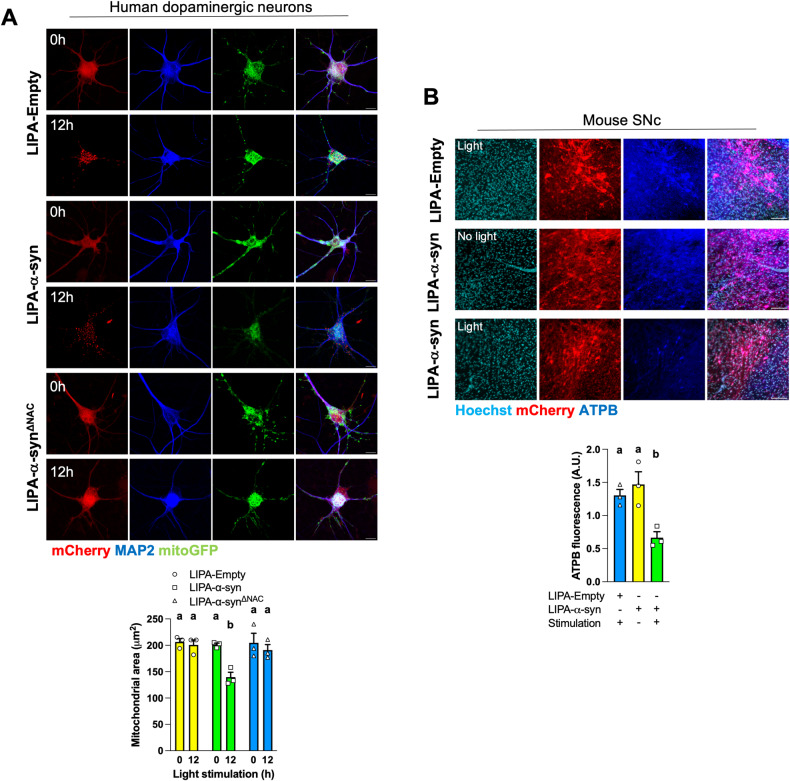
LIPA-induced α-syn aggregates decrease mitochondrial mass in human dopaminergic neuron and mouse midbrain. **A** Representative micrographs of human dopaminergic neurons expressing LIPA constructs and mitoGFP. Quantification of mitoGFP staining showing the decreased mitochondrial content upon stimulation of LIPA-α-syn (*n* = 3). Scale bar: 10 μm. **B** Representative micrographs of mouse SNc expressing LIPA constructs and stimulated as indicated. Scale bar: 100 μm. Quantification of ATPB fluorescence normalized by Hoechst staining (*n* = 3). Data are mean ± s.e.m., and were analyzed by two-way ANOVA (**A**) and by one-way ANOVA (**B**) followed by Tukey post-hoc tests. Data points with different letters are statistically different (*p* < 0.05).

Knowing that mammalian cell lines may not accurately replicate neuronal responses, we generated induced pluripotent stem cell (iPSC)-derived human dopaminergic neurons [32] co-expressing LIPA constructs and mitoGFP to examine the impact of LIPA-α-syn aggregates on mitochondria. Strikingly, the area covered by mitoGFP decreased after light stimulation of dopaminergic neurons expressing LIPA-α-syn but not in neurons expressing LIPA-Empty or LIPA-α-syn^ΔNAC^ (Fig. 4A).

We also analyzed how LIPA constructs would impact on the mitochondrial content in mouse SNc considering that in vivo activation of LIPA-α-syn within mouse SNc leads to dopaminergic neurodegeneration and PD-like symptoms [32]. Thus, AAVs encoding LIPA-Empty and LIPA-α-syn were stereotactically injected in mouse SNc. To stimulate the LIPA constructs in vivo, wireless optogenetic devices were also inserted in the SNc. After recovery and eight weeks of light stimulation, brains were extracted and processed, as described [32], to analyze the mitochondrial content. Like cell lines and human dopaminergic neurons, in vivo stimulation of LIPA-α-syn led to lower ATPB staining in the SNc as compared to unstimulated LIPA-α-syn and stimulated LIPA-Empty (Fig. 4B). Hence, the stimulation of LIPA-α-syn leads to lower mitochondrial content not only in mitotic cell lines but also in human dopaminergic neurons and mouse SNc.

### LIPA-α-syn aggregates disrupt mitochondrial morphology

**Fig. 5:**
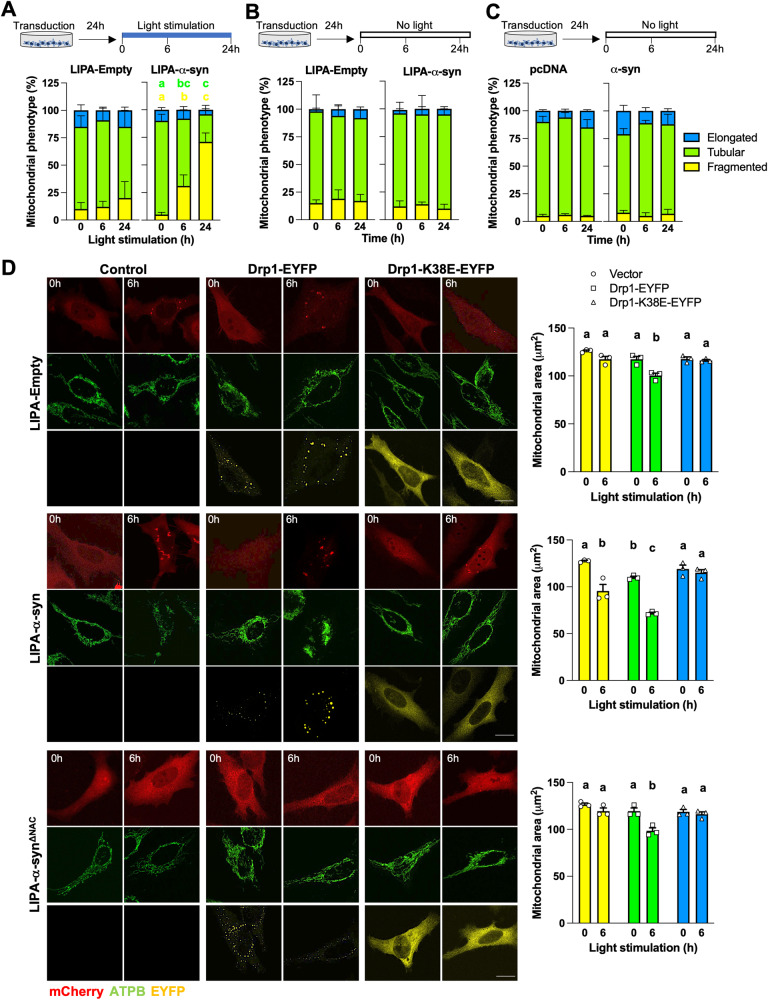
LIPA-induced α-syn aggregates induce Drp1-mediated mitochondrial fragmentation. **A** Quantification of the shape of the mitochondrial network in HEK cells expressing the LIPA constructs and stimulated as indicated (*n* = 3). **B** Quantification of the shape of the mitochondrial network in unstimulated HEK cells expressing the LIPA constructs and imaged as indicated (*n* = 3). **C** Quantification of the shape of the mitochondrial network in HEK cells expressing pcDNA or α-syn and imaged as indicated (*n* = 3). **D** Representative micrographs of HeLa cells stimulated as indicated and co-expressing LIPA constructs, Drp1-EYFP or the dominant-negative mutant Drp1-K38E-EYFP. The area covered by ATPB staining was quantified as a proxy for the total mitochondrial content. Scale bar: 20 μm. Data are mean ± s.e.m., and were analyzed by two-way ANOVA followed by Tukey post-hoc tests. Data points with different letters are statistically different (*p* < 0.05). In A, green letters indicate differences between tubular networks, and yellow letters indicate differences between fragmented networks. No other statistical difference were observed in (**A**, **B**, **C**).

Multiple reports showed that overexpression of α-syn induce mitochondrial fragmentation [21]. The impact of α-syn overexpression and aggregation on mitochondrial morphology was then assessed in three different conditions. For this, cells were classified according the morphology of their mitochondrial network [37, 38]. The mitochondrial network was classified as fragmented when mitochondria were short and spherical; hyperfused when more than 50% of mitochondria were longer than 5 μm and highly interconnected; tubular when the mitochondrial network appeared as an intermediate between fragmented and elongated. The number of cells with fragmented mitochondria increased, and the number of cells with tubular mitochondria decreased over time in stimulated HeLa cells expressing LIPA-α-syn but not in cells expressing LIPA-Empty (Fig. 5A). Interestingly, no change of mitochondrial shape was detected in unstimulated cells (Fig. 5B) or cells expressing α-syn alone (Fig. 5C). These findings suggest that the aggregation of α-syn is necessary for the well-known α-syn-induced mitochondrial fragmentation.

To examine whether the mitochondrial fission machinery is involved in the degradation of the mitochondria induced by LIPA-α-syn aggregation, we co-expressed Drp1-EYFP and the dominant-negative Drp1-K38E-EYFP (which blocks Drp1-mediated mitochondrial fission [43]) with LIPA constructs. Interestingly, expression of Drp1-EYFP increased the loss of mitochondria in HeLa cells expressing LIPA-α-syn as compared to cells co-expressing the control vector (Fig. 5D). Mitochondrial loss upon blue light stimulation was also observed in cells expressing LIPA-Empty or LIPA-α-syn^ΔNAC^ (Fig. 5D), suggesting that Drp1 overexpression sensitizes cells towards mitochondrial degradation independently of α-syn aggregation. Nevertheless, expression of the dominant negative Drp1-K38E-EYFP completely abolished the mitochondrial degradation induced by α-syn aggregation (Fig. 5D), indicating that the pro-fission activity of Drp1 is necessary for the fragmentation and degradation of mitochondria induced by LIPA-α-syn aggregates.

### LIPA-induced α-syn aggregates degrade mitochondria via autophagy

**Fig. 6:**
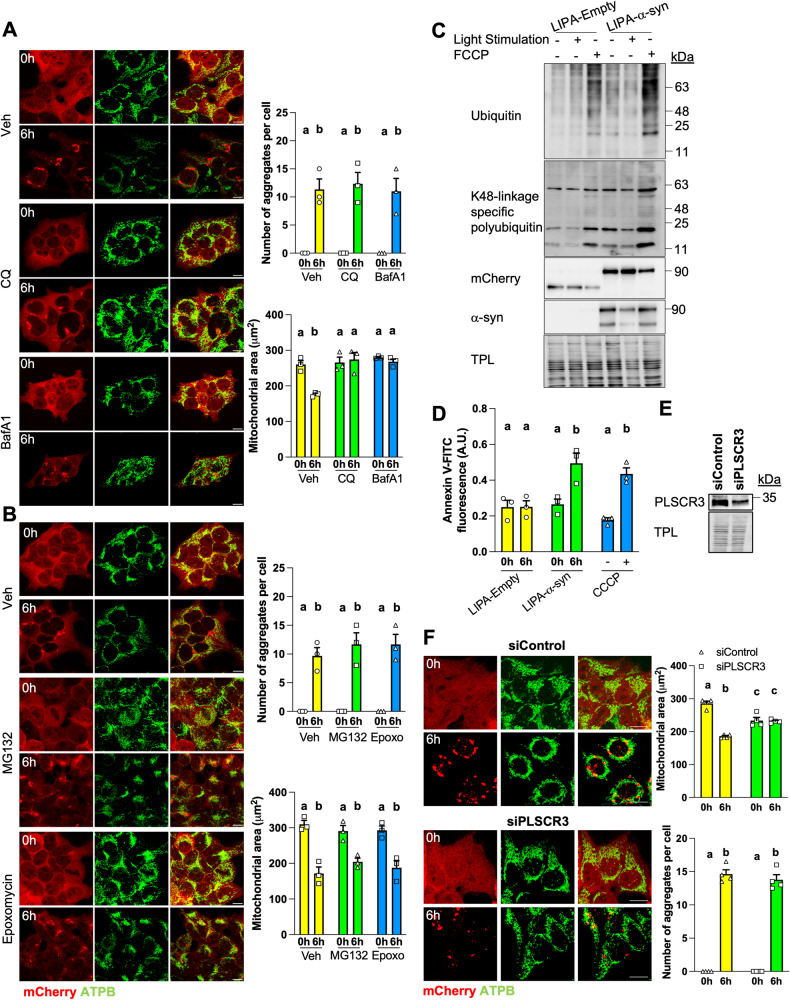
LIPA-α-syn aggregates trigger mitophagy via cardiolipin externalization. **A** Representative micrographs and quantification of HEK cells expressing LIPA-α-syn, stimulated as indicated, and treated with vehicle (Veh), chloroquine (CQ, 6 h, 25 μM) or bafilomycin A1 (BafA1, 6 h, 100 nM) and immunolabeled for ATPB. The area covered by ATPB staining was quantified as a proxy for the total mitochondrial content (*n* = 3). **B** Representative micrographs and quantification in HEK cells expressing LIPA-α-syn, stimulated as indicated, treated with vehicle (Veh), MG132 (6 h, 25 μM) or epoxomycin (6 h, 2.5 μM) and immunolabeled for ATPB (*n* = 3). **C** Immunoblotting of ubiquitin and K48-linkage specific polyubiquitin on mitochondria isolated from HEK cells expressing the LIPA constructs, treated and stimulated (during 6 h) as indicated (*n* = 3). TPL: total protein load. **D** Cardiolipin exposed to the surface of mitochondria isolated from HEK cells expressing LIPA constructs or from mock cells treated with CCCP (2 μM for 1 h) was labeled with Annexin-V-FITC and quantified (*n* = 3). **E** Representative immunoblotting (*n* = 3) of PLSCR3 in HEK cells upon treatment with siControl or siPLSCR3. **F** Representative micrographs and quantification (*n* = 4) of HEK cells expressing LIPA-α-syn and treated as indicated, showing that silencing of PLSCR3 blocks the degradation of mitochondria induced by LIPA-α-syn aggregates. Scale bar: 10 μm. Data are mean ± s.e.m., and were analyzed by two-way ANOVA followed by Tukey post-hoc tests. Data points with different letters are statistically different (*p* < 0.05).

To identify the pathway involved in the degradation of mitochondria induced by the LIPA-dependent aggregation, HEK^LIPA-α-syn^ were first treated with inhibitors of autophagy. Chloroquine and bafilomycin A1 did not reduce the number of aggregates but completely blocked the degradation of mitochondria induced by LIPA-α-syn aggregation (Fig. 6A). The two autophagy inhibitors also blocked the mitochondrial degradation induced by LIPA-α-syn aggregates in HeLa and N2A cells (Fig. S5A, B). Mitochondrial proteins can also be selectively degraded via the ubiquitin-proteasome system [44]. However, treatment with the inhibitors of the proteasome MG132 and epoxomicin had no impact on the number of aggregates and the degradation of mitochondria induced by α-syn aggregation in HEK^LIPA-α-syn^ (Fig. 6B) and in HeLa cells expressing LIPA-α-syn (Fig. S6C), suggesting that mitochondria are degraded by mitophagy after induction of α-syn aggregation.

Multiple pathways are involved in the degradation of mitochondria during mitophagy, including ubiquitin-dependent and -independent mechanisms. For instance, ubiquitylation of mitochondrial proteins can serve as signal to recruit autophagic receptors such as p62 or NDP52 [44]. We thus examined ubiquitylation of mitochondrial proteins during stimulation of the LIPA constructs. Surprisingly, ubiquitylation and K48-linked polyubiquitylation (the canonical signal for protein degradation by the proteasome [45]) of mitochondrial proteins did not increase after light stimulation in both HEK^LIPA-Εmpty^ and HEK^LIPA-α-syn^, unlike after treatment with the uncoupler FCCP (Fig. 6C). Moreover, overexpression of ubiquitin or ubiquitin-KO (in which all lysine residues have been changed for arginine and are thus unable to form ubiquitin chain [46]) in HeLa and N2A cells expressing LIPA-α-syn did not impact the mitochondrial degradation induced by α-syn aggregation (Fig. S5D). These findings suggest that α-syn aggregation triggers degradation of mitochondria via ubiquitin-independent mitophagy.

During ubiquitin-independent mitophagy, different receptors localized on the mitochondrial outer membrane can directly interact with LC3 to trigger the elimination of mitochondria [44, 47]. For instance, cardiolipin can translocate from the inner mitochondrial membrane to the outer mitochondrial membrane and directly bind LC3 in autophagosome upon pro-mitophagy stimuli [48]. To examine whether this process is involved in the mitochondrial degradation induced by LIPA-α-syn aggregates, mitochondria were isolated from HEK^LIPA-Εmpty^, HEK^LIPA-α-syn^ and from mock cells treated with FCCP (which promotes cardiolipin externalization [48]). Then, mitochondria were stained with Annexin-V-FITC to monitor cardiolipin exposed at the surface of the organelles, as described [41, 48, 49]. Strikingly, labeling with Annexin-V was increased in mitochondria isolated from stimulated HEK^LIPA-α-syn^ and cells treated FCCP, but not in mitochondria isolated from stimulated HEK^LIPA-Εmpty^ (Fig. 6D). To further test the role of cardiolipin externalization, phospholipid scramblase 3 (the enzyme translocating cardiolipin to mitochondrial surface) was downregulated using siRNA (Fig. 6E). Silencing of phospholipid scramblase 3 had no impact on the number of LIPA-α-syn aggregates but completely abolished the degradation of mitochondria in stimulated HEK^LIPA-α-syn^ (Fig. 6E, F) and HeLa cells expressing LIPA-α-syn (Fig. S6A). BNIP3L and FUNDC1 localized at the surface of mitochondria can also bind to LC3 to mediate mitophagy [47]. We thus examined whether these receptors could be involved in the mitophagy induced by LIPA-dependent α-syn aggregation. Neither siBNIP3L nor siFUNDC1 prevented the degradation of mitochondria induced by α-syn aggregation (Fig. S6B, C). Therefore, we suggest that LIPA-α-syn aggregates impair mitochondrial physiology, leading to transfer of cardiolipin to the mitochondrial surface, and consequently, to mitophagy.

## Discussion

The aim of this work was to characterize the specific impact of α-syn aggregation on mitochondria using a light-induced protein aggregation system, recently developed by our team, and allowing for the spatiotemporal control the aggregation of α-syn [32]. We demonstrate that the LIPA-induced clustering of α-syn induces mitochondrial depolarization, lower cellular respiration and ATP production, resulting in Drp1-mediated mitochondrial fragmentation, and mitophagy via cardiolipin externalization. Overexpression of α-syn alone did not generate aggregates and did not alter mitochondria, demonstrating that aggregation is necessary for α-syn to impair mitochondria. Thus, LIPA constructs represent appropriate tools to decipher between the impact of α-syn aggregation and overexpression and will be likely beneficial to identify new therapeutic strategies for the treatment of PD and related disorders.

Multiplication of α-syn gene copies is linked to PD [50], suggesting that an increase in α-syn expression is sufficient to induce its aggregation and cause degeneration. For instance, doubling α-syn expression is sufficient to induce cytoplasmic inclusions in yeast [51]. However, aggregates are not systematically observed or well characterized upon α-syn overexpression in other models [25–30, 52]. The present work supports that increased expression of α-syn does not always lead to its aggregation, at least in our experimental conditions, and that α-syn could induce cellular defects independently of its aggregation. In this context, the LIPA constructs allowed us to demonstrate that the aggregation of α-syn is necessary to induce alterations of mitochondria.

In our aggregation system, oligomers and large aggregates were visible by immunoblotting and standard confocal microscopy as early as after 3 h of stimulation of LIPA-α-syn. We previously observed that α-syn fibrils can be detected after 6 h of stimulation [32]. The large α-syn aggregates visible by microscopy become positive for several markers of Lewy bodies after 12 h of stimulation, including phosphorylated α-syn (pS129), ubiquitin, thioflavin S, p62 and HSP70 [32]. Although fibrils and these markers were not examined at earlier time points, we can suggest that mitochondria are first altered by their interactions with α-syn oligomers and fibrils.

The LIPA-dependent aggregation of α-syn disrupted OXPHOS. How α-syn clusters can disrupts mitochondrial metabolism remains undetermined. It is possible that α-syn aggregates bind directly and physically with specific mitochondrial proteins to alter their functional properties. Several mitochondrial proteins were shown to physically interact with α-syn although it is not clear whether these interactions are specific for α-syn oligomers or monomers. For instance, interaction between TOM20 and α-syn was detected by proximity ligation assay in HEK293 cells treated with oligomeric α-syn, which was associated with damped importation of nuclear-encoded mitochondrial proteins and lower cellular respiration [22]. In rat co-culture of neurons and astrocytes, oligomers of recombinant α-syn were shown to interact with ATP synthase subunit α and TOM20 by colocalization and proximity ligation assays leading to mitochondrial depolarization and the opening of the permeability transition pore [53]. Indirect evidence also suggests that α-syn oligomers interact with cardiolipin to favor pore formation within mitochondrial membranes [54–56]. Considering that antibodies for α-syn poorly discriminate between monomers, oligomers and fibrils [57], it is still uncertain which α-syn species physically interact with mitochondria. Here, we observed little overlap between unstimulated LIPA-α-syn and mitochondria and brief kiss-and-run contacts between the organelle and LIPA-α-syn aggregates. Thus, we suggest that the LIPA-induced aggregates of α-syn are not imported within mitochondria and rather contact with the cytosolic face of mitochondria. LIPA-induced aggregates of α-syn could trigger mitochondrial dysfunctions via direct interactions with specific domains of mitochondrial outer membranes. Oligomers of α-syn can bind to cardiolipin [54, 58], which can represent up to 5% of lipids of the outer mitochondrial membrane [59], leading to pore formation and mitochondrial dysfunction [54]. Localization of α-syn at MAMs was also reported in different models [23, 24, 60, 61]. Several studies showed that overexpression of α-syn disrupted the physical contacts and Ca^2+^ transfer between the two organelles, leading to alterations of mitochondrial metabolism [23, 24, 61]. LIPA-induced aggregates of α-syn could thus directly disrupt mitochondrial metabolism via interactions with cardiolipin-rich microdomains or contact sites between mitochondria and ER.

Our findings suggest that the depolarization of mitochondria resulted in the translocation of cardiolipin to the outer mitochondrial membrane and consequently, mitophagy. There are various pathways through which mitochondria can be degraded during mitophagy, and the PINK1-Parkin pathway was one the first described. Briefly, PINK1 is constitutively degraded in healthy mitochondria. In depolarized mitochondria, PINK1 accumulate and recruit cytosolic Parkin to the outer mitochondrial membrane [62]. In turn, Parkin ubiquitinylates several mitochondrial proteins and facilitates the recruitment of autophagy receptors including p62 [63, 64]. Considering that HeLa cells lack fully functional Parkin [65], and that overexpression of ubiquitin unable to form polyubiquitin chain did not blocked degradation of mitochondria upon aggregation of α-syn, we suggest that LIPA-induced aggregates of α-syn could not induce mitophagy through the PINK1-Parkin pathway, at least in HeLa cells and/or in our specific experimental conditions. In fact, LIPA-α-syn aggregates promoted cardiolipin externalization at the mitochondrial surface, and the blockade of cardiolipin transport to the outer mitochondrial membrane by siPLSCR3 prevented degradation of mitochondria upon by LIPA-induced aggregation of α-syn. Cardiolipin externalization was previously observed in human DA neurons expressing the A53T mutant of *SNCA* [66], upon association of monomeric α-syn with mitochondria but before any mitochondrial dysfunctions could be noted [66]. The same study showed that α-syn competed with LC3 for binding with vesicles mimicking the outer mitochondrial membrane, suggesting the presence of α-syn would prevent clearance of mitochondria by autophagy. Moreover, cardiolipin localized at mitochondrial surface can buffer the toxic effects of α-syn since it promotes refolding of pathological oligomers and fibrils into monomers [66]. Nevertheless, our findings indicate that cardiolipin externalization was triggered after the aggregation of α-syn, in parallel to mitochondrial alterations and was necessary for the LIPA-induced mitochondrial clearance by autophagy.

In conclusion, the present study demonstrates that the aggregation of α-syn by the LIPA system triggers disruption of OXPHOS, leading to Drp1-mediated mitochondrial fission and cardiolipin externalization-dependent mitophagy. Our novel LIPA tools enabling the spatiotemporal control of α-syn aggregation will help to identify therapeutic targets in PD in future studies.

## Availability of data and material

Complete data or material used in the present study will be available upon reasonable request.

### Reporting summary

Further information on research design is available in the Nature Research Reporting Summary linked to this article.

### Supplementary information

original data files

S1

S2

S3

S4

S5

S6

Figures legends for supplementary figures

Video 1. Interaction between LIPA-Empty aggregates and mitochondria in unstimulated HEK<sup>LIPA-Empty</sup>.

Video 2. Interaction between LIPA-Empty aggregates and mitochondria in HEK<sup>LIPA-Empty</sup> stimulated for 3h

Video 3. Interaction between LIPA-Empty aggregates and mitochondria in HEK<sup>LIPA-Empty</sup> stimulated for 6h

Video 4. Interaction between LIPA-Empty aggregates and mitochondria in HEK<sup>LIPA-Empty</sup> stimulated for 12h

Video 5. Interaction between LIPA-Empty aggregates and mitochondria in HEK<sup>LIPA-Empty</sup> stimulated for 24h

Video 6. Interaction between LIPA-α-syn aggregates and mitochondria in unstimulated HEK<sup>LIPA-α-syn</sup>

Video 7. Interaction between LIPA-α-syn aggregates and mitochondria in HEK<sup>LIPA-α-syn</sup> stimulated for 3h

Video 8. Interaction between LIPA-α-syn aggregates and mitochondria in HEK<sup>LIPA-α-syn</sup> stimulated for 6h

Video 9. Interaction between LIPA-α-syn aggregates and mitochondria in HEK<sup>LIPA-α-syn</sup> stimulated for 12h

Video 10. Interaction between LIPA-α-syn aggregates and mitochondria in HEK<sup>LIPA-α-syn</sup> stimulated for 24h

Video S1. Interaction between LIPA-Empty aggregates and mitochondria in live HeLa cells after 2 min stimulation

Video S2. Interaction between LIPA-Empty aggregates and mitochondria in live HeLa cells after 6h stimulation

Video S3. Interaction between LIPA-Empty aggregates and mitochondria in live HeLa cells after 24h stimulation

Video S4. Interaction between LIPA-α-syn aggregates and mitochondria in live HeLa cells after 2 min stimulation

Video S5. Interaction between LIPA-α-syn aggregates and mitochondria in live HeLa cells after 6h stimulation

Video S6. Interaction between LIPA-α-syn aggregates and mitochondria in live HeLa cells after 24h stimulation

Reporting Summary

Videos 1–10
