# Supplementary figures and images for: Aggregation of alpha-synuclein disrupts mitochondrial metabolism and induce mitophagy via cardiolipin externalization
Source: Cell Death Dis. 2023 Nov 10;14(11):729. doi: 10.1038/s41419-023-06251-8 (PMC10638290; doi:10.1038/s41419-023-06251-8)

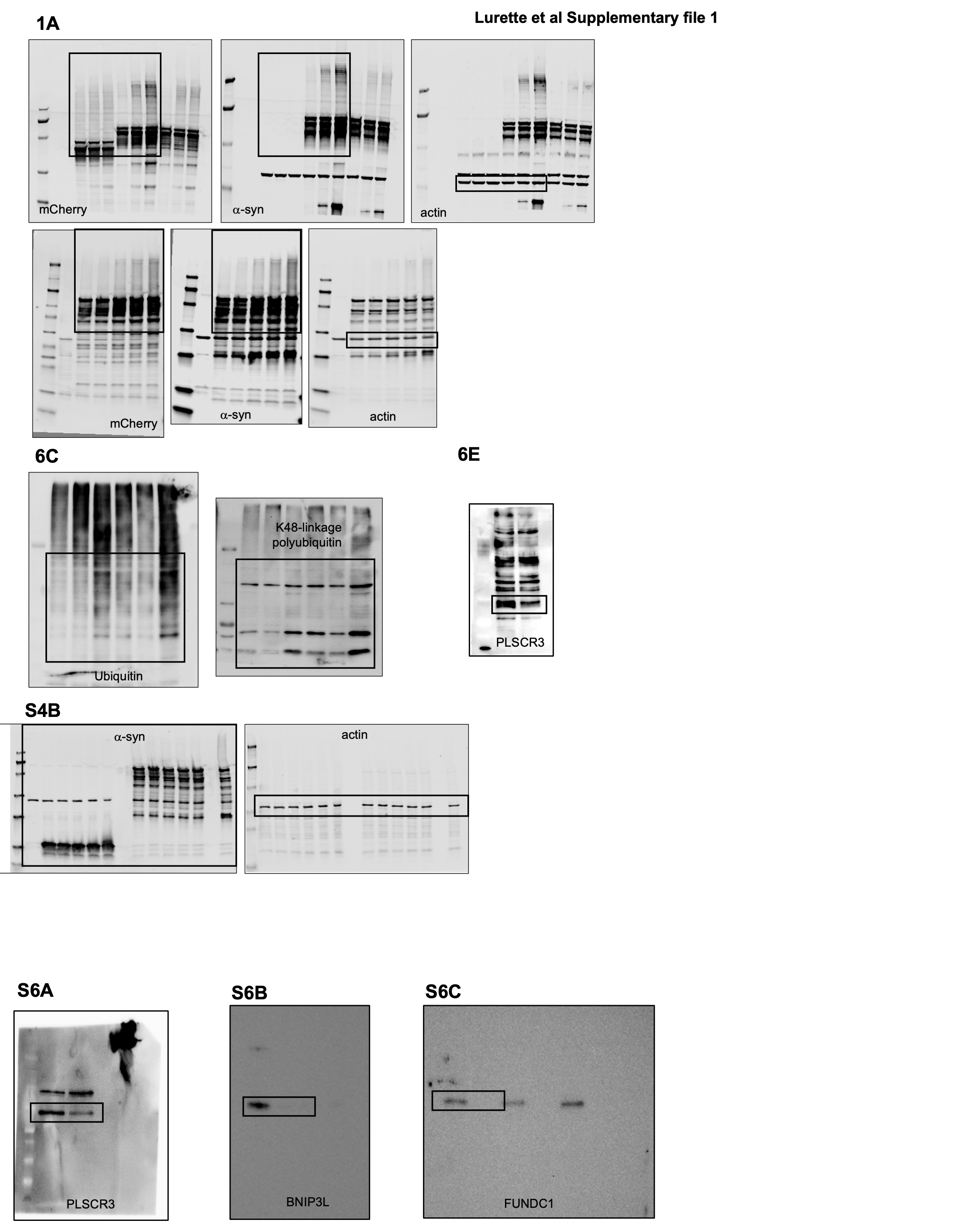

Supplement: Supplementary file 1 — original data files [file 41419_2023_6251_MOESM1_ESM.tif]

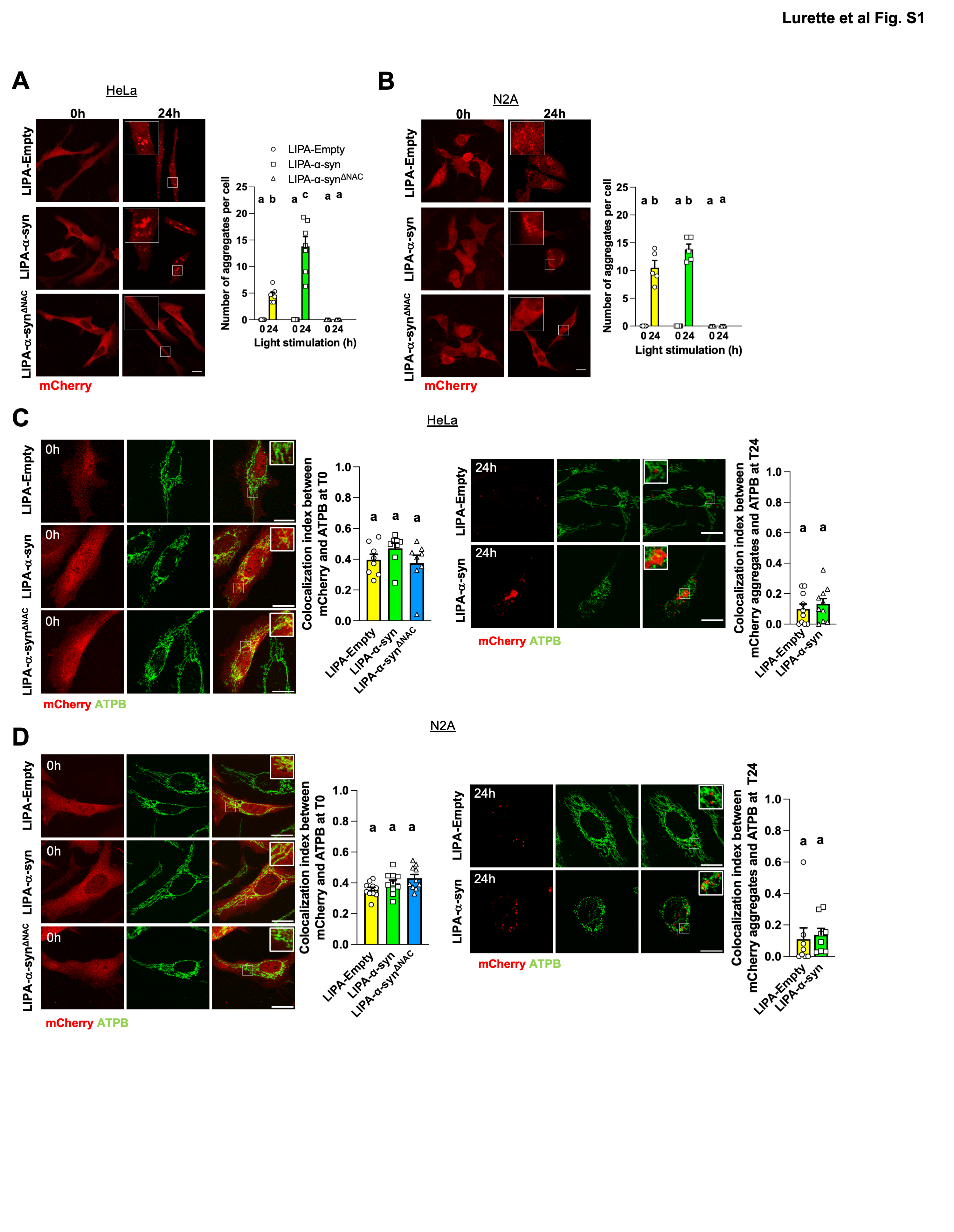

Supplement: Supplementary file 2 — S1 [file 41419_2023_6251_MOESM2_ESM.tif]

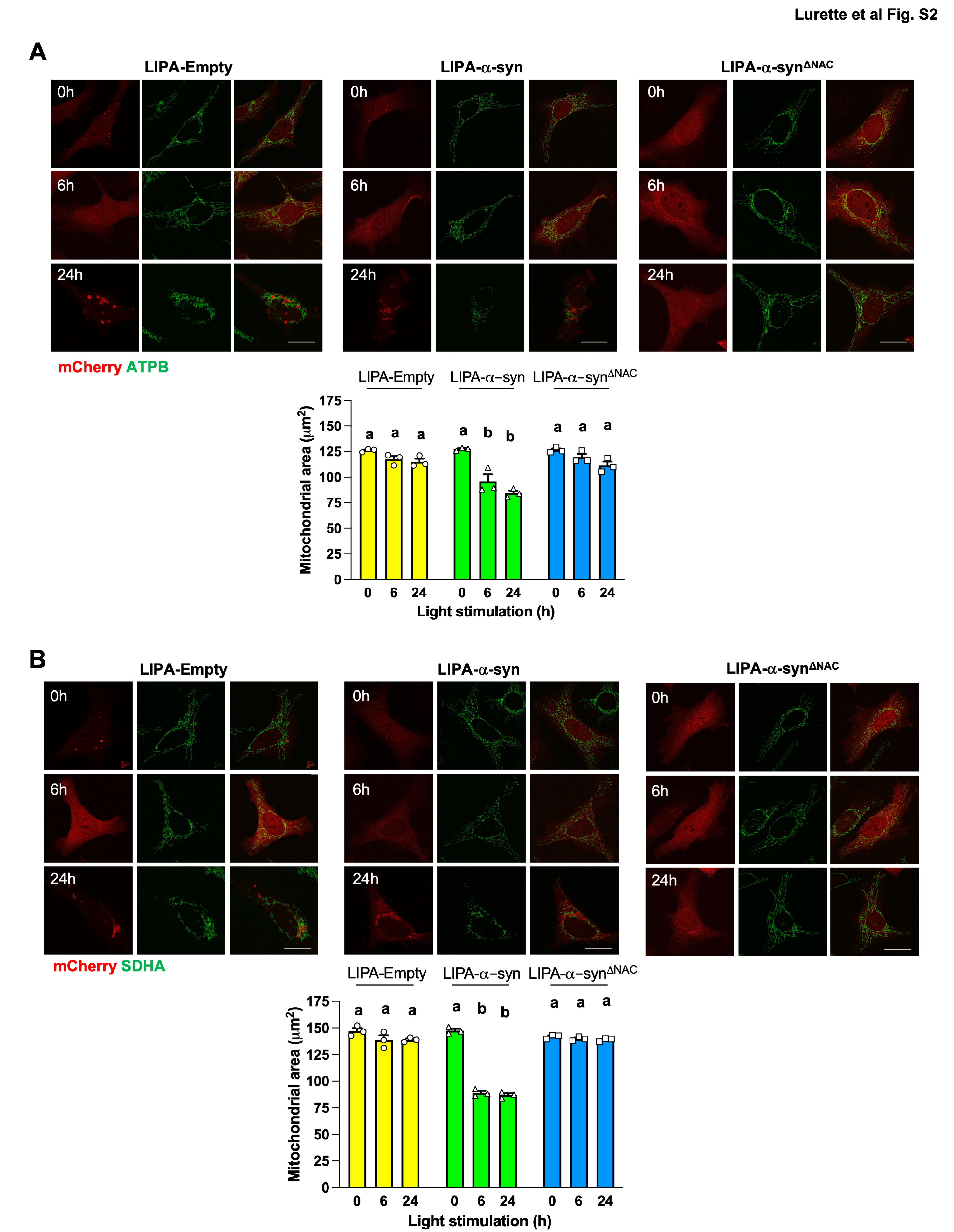

Supplement: Supplementary file 3 — S2 [file 41419_2023_6251_MOESM3_ESM.tif]

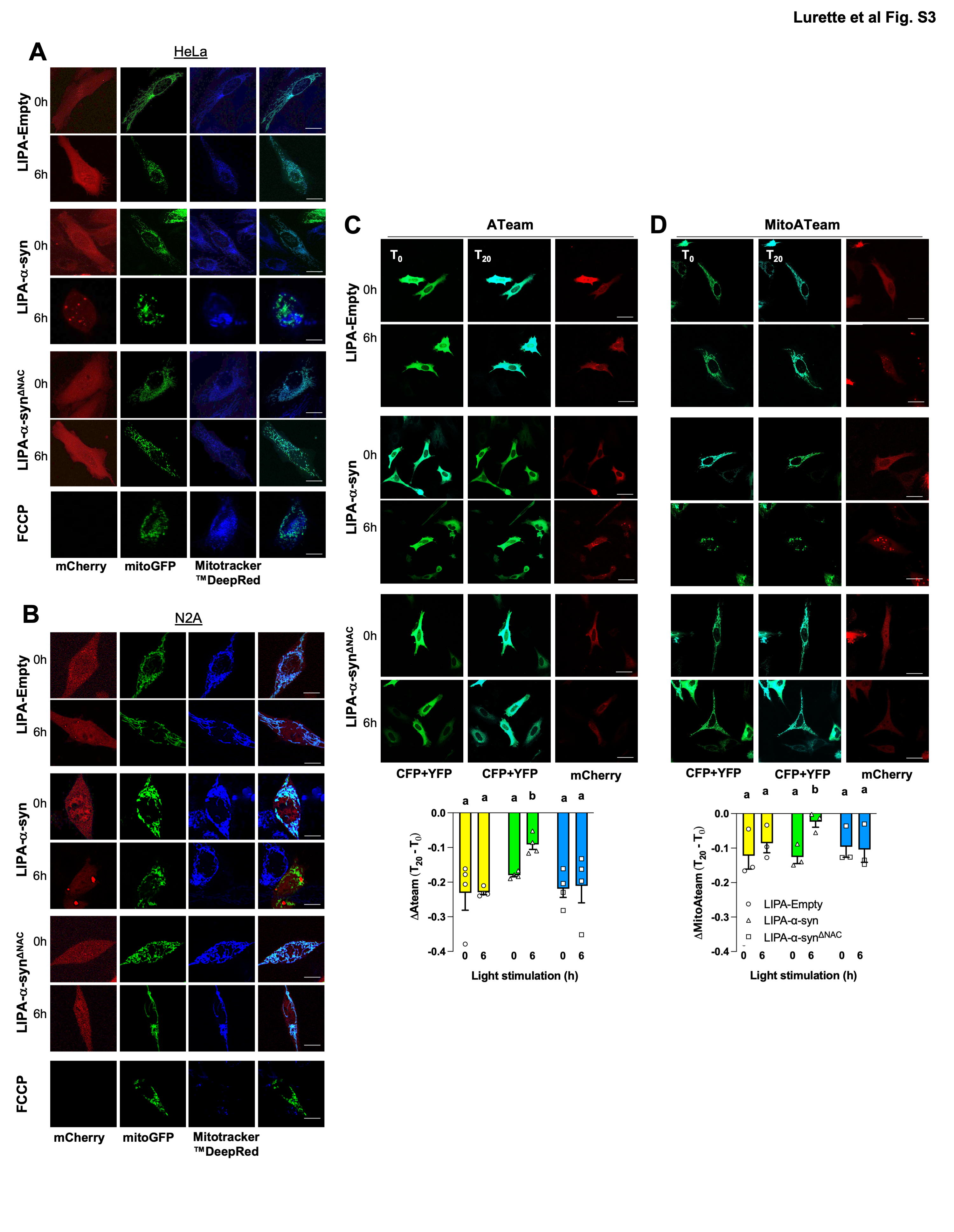

Supplement: Supplementary file 4 — S3 [file 41419_2023_6251_MOESM4_ESM.tif]

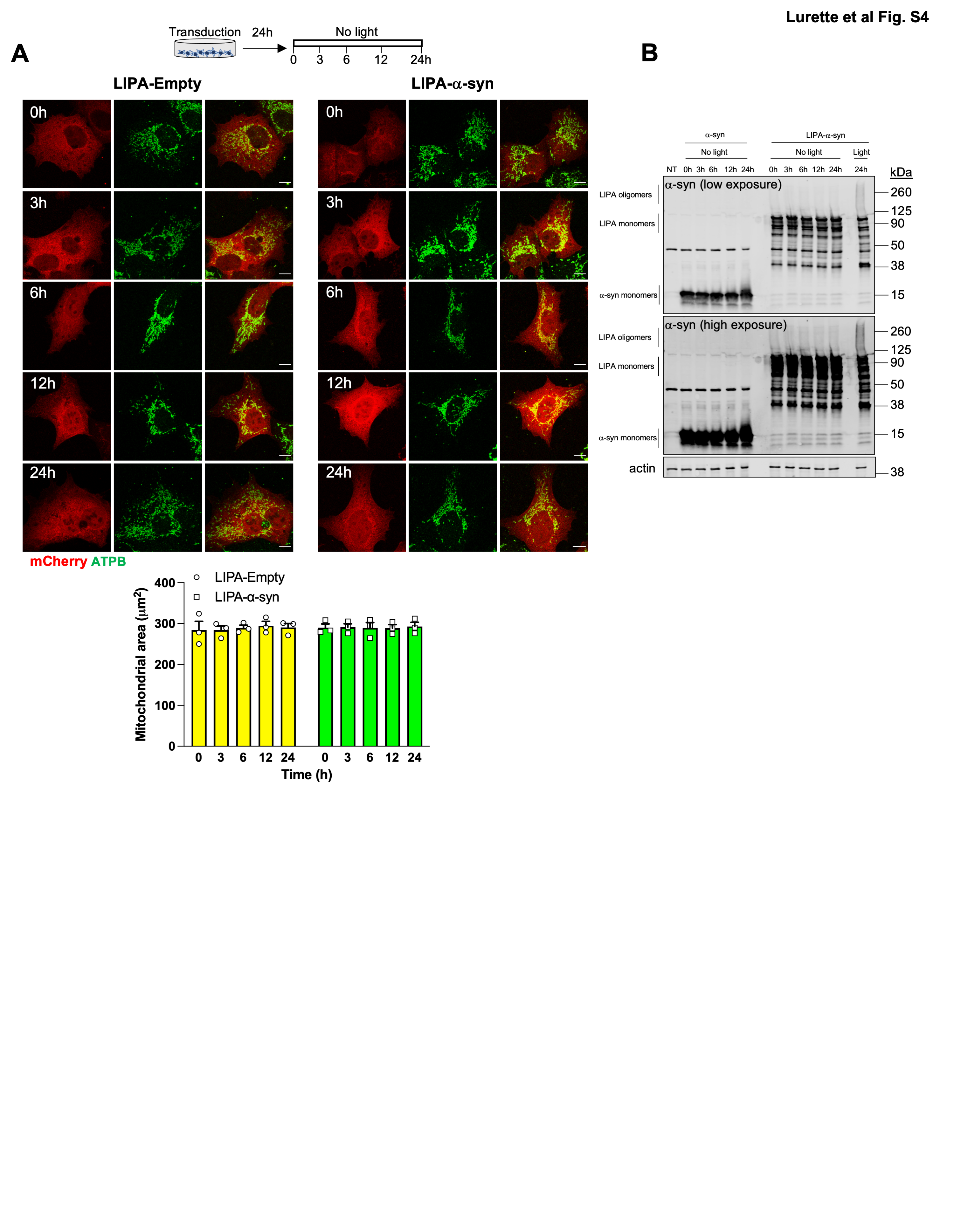

Supplement: Supplementary file 5 — S4 [file 41419_2023_6251_MOESM5_ESM.tif]

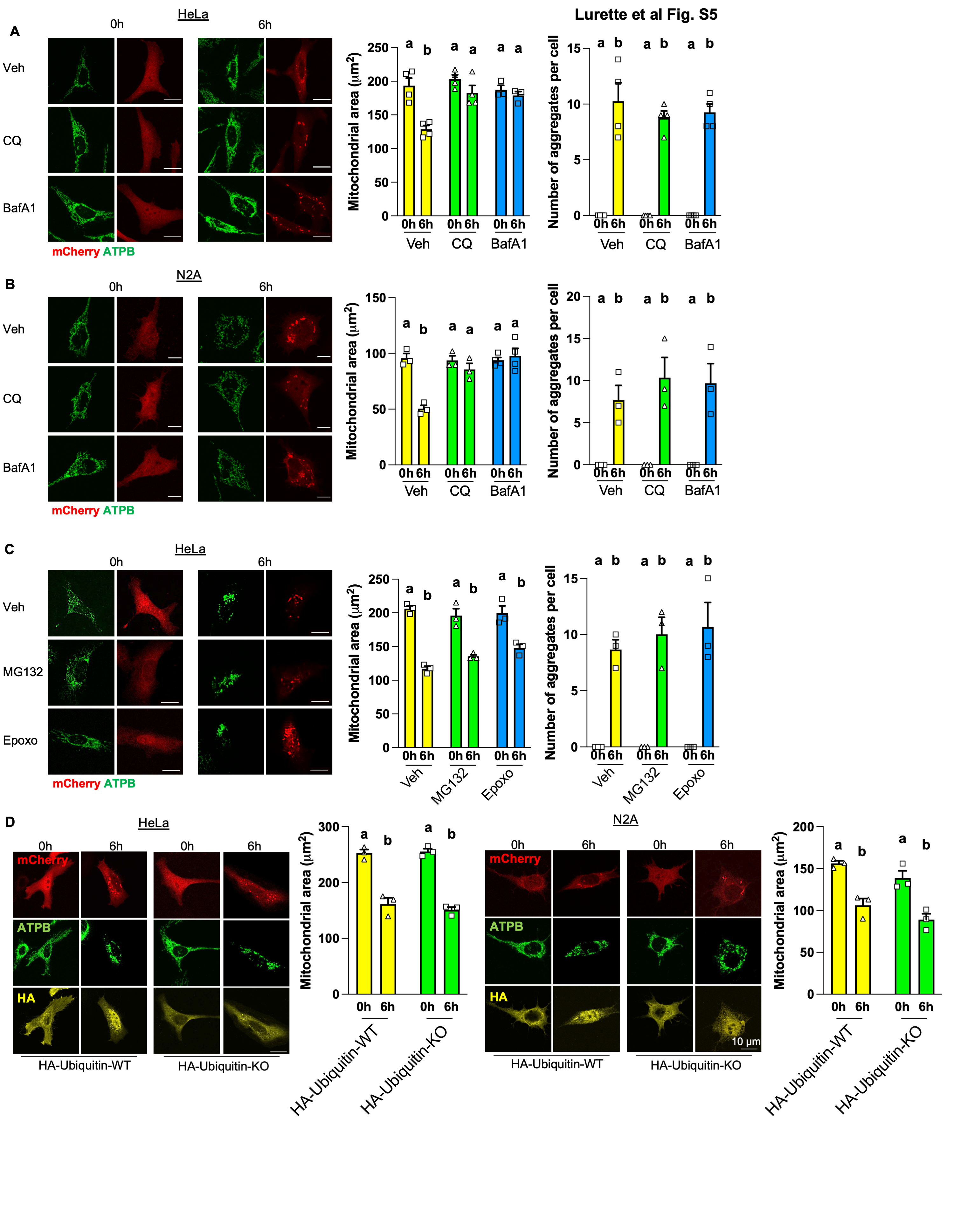

Supplement: Supplementary file 6 — S5 [file 41419_2023_6251_MOESM6_ESM.tif]

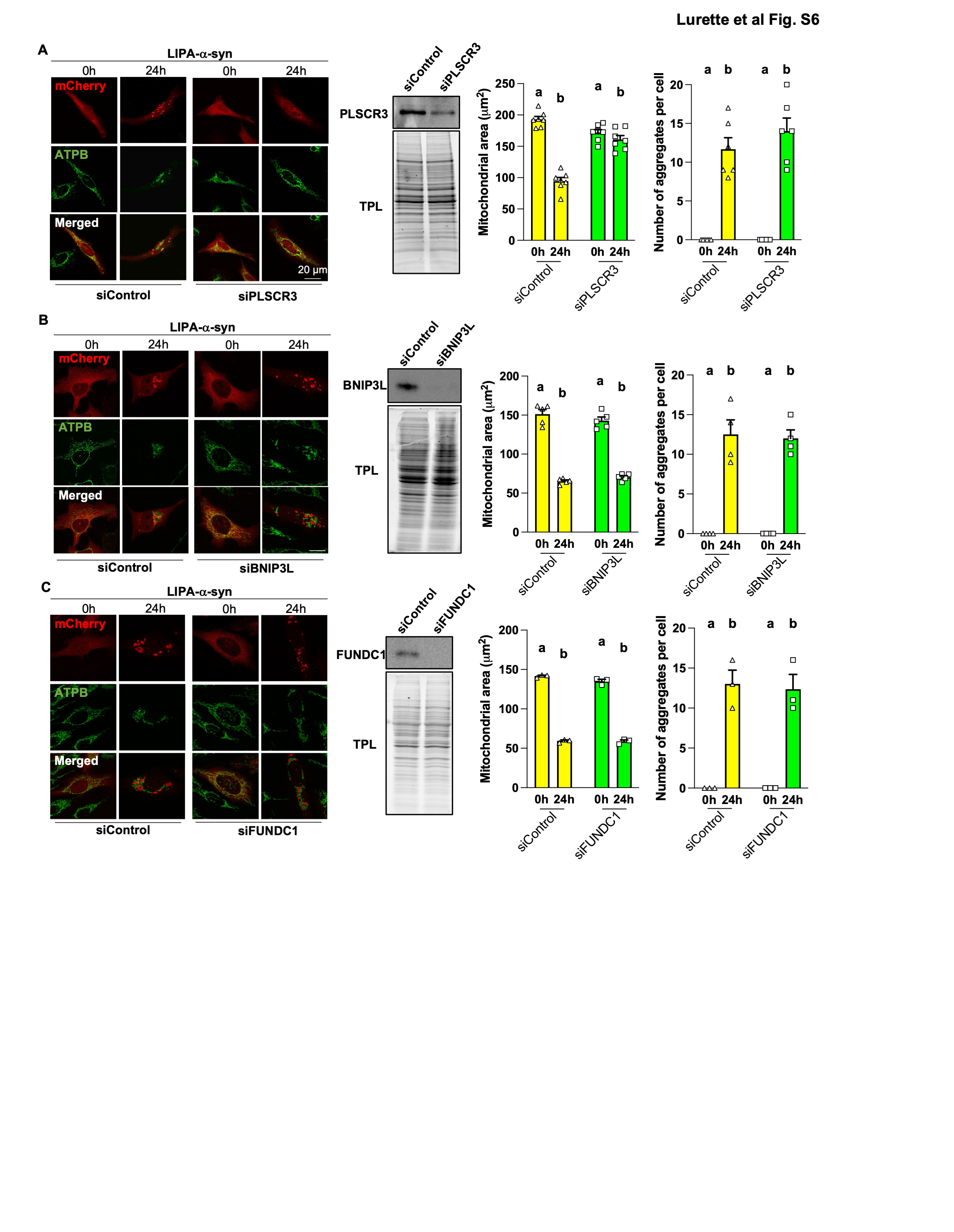

Supplement: Supplementary file 7 — S6 [file 41419_2023_6251_MOESM7_ESM.tif]
