# Supplementary material for: Aggregation of alpha-synuclein disrupts mitochondrial metabolism and induce mitophagy via cardiolipin externalization
Source: Cell Death Dis. 2023 Nov 10;14(11):729. doi: 10.1038/s41419-023-06251-8 (PMC10638290; doi:10.1038/s41419-023-06251-8)
Supplement: Supplementary file 8 — Figures legends for supplementary figures [file 41419_2023_6251_MOESM8_ESM.docx]

**Supplementary information**

**Figure S1.** (**A-B**) Representative micrographs and quantification (n=5-6) showing number of aggregates in HeLa (A) and N2A (B) cells expressing LIPA constructs and stimulated as indicated. (**C-D**) Representative micrographs and Pearson’s colocalization index between mCherry and ATPB in HeLa (C) or N2A (D) cells expressing LIPA constructs and stimulated as indicated (n=8-10). Scale bar for HeLa: 20 μm; Scale bar for N2A: 10 μm. Data are mean ± s.e.m., and were analyzed by two-way ANOVA followed by Tukey post-hoc tests, except for colocalization analysis in stimulated cells where data were analyzed by Student t-test. Data points with different letters are statistically different (p < 0.05).

**Figure S2.** (**A-B**) Representative micrographs (n=3) of HeLa cells expressing LIPA constructs, stimulated as indicated and stained for the mitochondrial proteins ATPB (A) and SDHA (B). The area covered by ATPB or SDHA staining was quantified as a proxy for the total mitochondrial content. Scale bar for HeLa: 20 μm. Data are mean ± s.e.m., and were analyzed by two-way ANOVA followed by Tukey post-hoc tests. Data points with different letters are statistically different (p < 0.05).

**Figure S3.** (**A-B**) Representative micrographs of HeLa (A) or N2A (B) cells expressing LIPA constructs and stained with MitoTracker Deep Red™ or mock cells treated with the uncoupler FCCP. (**C-D**) Representative micrographs and quantification of HeLa cells co-expressing LIPA constructs and ATeam (C) or mitoATeam (D) constructs, stimulated as indicated, before (T_0_) and after (T_20_) treatment with 2-deoxyglucose (20 mM) and KCN (1 mM) (n=3-4). Scale bar for HeLa: 20 μm; Scale bar for N2A: 10 μm. Data are mean ± s.e.m., and were analyzed by two-way ANOVA followed by Tukey post-hoc tests. Data points with different letters are statistically different (p < 0.05).

**Figure S4.** (**A**) Representative micrographs of HEK cells expressing LIPA constructs and imaged as indicated without light stimulation, and immunolabeled for the mitochondrial protein ATPB. The area covered by ATPB staining was quantified as a proxy for the total mitochondrial content (n=3). Scale bar: 10 μm. (**B**) Representative immunoblotting (n=3) of α-syn and actin in HEK cells expressing α-syn or LIPA-α-syn and exposed to blue light as indicated. NT: non-treated. Data are mean ± s.e.m., and were analyzed by two-way ANOVA followed by Tukey post-hoc tests. Data points with different letters are statistically different (p < 0.05).

**Figure S5.** (**A-B**) Representative micrographs of HeLa (A) and N2A (B) cells expressing LIPA-α-syn, stimulated as indicated, treated with chloroquine (CQ, 6h, 60 μM) or bafilomycin A1 (BafA1, 6h, 100 nM) and immunolabeled for ATPB. The area covered by ATPB staining was quantified as a proxy for the total mitochondrial content (n=3-4). (**C**) Representative micrographs and quantification of ATPB in HeLa cells expressing LIPA-α-syn, stimulated as indicated, treated with MG132 (6h, 25 μM) or epoxomycin (6h, 2.5 μM) (n=3). (**D**) Representative micrographs and quantification of ATPB in HeLa or N2A cells stimulated as indicated, co-expressing LIPA-α-syn and HA-ubiquitin-WT or HA-ubiquitin-KO, and immunolabeled for ATPB (n=3). Scale bar for HeLa: 20 μm; Scale bar for N2A: 10 μm. Data are mean ± s.e.m., and were analyzed by two-way ANOVA followed by Tukey post-hoc tests. Data points with different letters are statistically different (p < 0.05).

**Figure S6.** (**A-C**) Representative micrographs and immunoblotting of HeLa cells expressing LIPA-α-syn, stimulated as indicated, and treated with siControl, siPLSCR3 (A), siBNIP3L (B) or siFUNDC1 (C). Quantification of the ATPB staining in HeLa cells treated as indicated (n=3-4). Scale bar: 20 μm. Data are mean ± s.e.m., and were analyzed by two-way ANOVA followed by Tukey post-hoc tests. Data points with different letters are statistically different (p < 0.05).

**Video S1. Interaction between LIPA-Empty aggregates and mitochondria in live HeLa cells after 2 min stimulation**. Live HeLa cells co-expressing LIPA-Empty and mitoGFP were stimulated for 2 min and imaged during 7,5 min.

**Video S2. Interaction between LIPA-Empty aggregates and mitochondria in live HeLa cells after 6h stimulation**. Live HeLa cells co-expressing LIPA-Empty and mitoGFP were stimulated for 6h and imaged during 7,5 min.

**Video S3. Interaction between LIPA-Empty aggregates and mitochondria in live HeLa cells after 24h stimulation**. Live HeLa cells co-expressing LIPA-Empty and mitoGFP were stimulated for 24h and imaged during 7,5 min.

**Video S4. Interaction between LIPA-α-syn aggregates and mitochondria in live HeLa cells after 2 min stimulation**. Live HeLa cells co-expressing LIPA-α-syn and mitoGFP were stimulated for 2 min and imaged during 7,5 min.

**Video S5. Interaction between LIPA-α-syn aggregates and mitochondria in live HeLa cells after 6h stimulation**. Live HeLa cells co-expressing LIPA-α-syn and mitoGFP were stimulated for 6h and imaged during 7,5 min.

**Video S6. Interaction between LIPA-α-syn aggregates and mitochondria in live HeLa cells after 24h stimulation**. Live HeLa cells co-expressing LIPA-α-syn and mitoGFP were stimulated for 24h and imaged during 7,5 min.

**Supplementary file 1. Uncropped immunoblottings.**
