# Supplementary material for: Aggregation of alpha-synuclein disrupts mitochondrial metabolism and induce mitophagy via cardiolipin externalization
Source: Cell Death Dis. 2023 Nov 10;14(11):729. doi: 10.1038/s41419-023-06251-8 (PMC10638290; doi:10.1038/s41419-023-06251-8)
Supplement: Supplementary file 26 — Videos 1–10 [file 41419_2023_6251_MOESM26_ESM.docx]

**Video 1.** **Interaction between LIPA-Empty** **aggregates and mitochondria in unstimulated HEK^LIPA-Empty^**. Live HEK cells co-expressing LIPA-Empty and mitoGFP were imaged during 5 min.

**Video 2.** **Interaction between LIPA-Empty** **aggregates and mitochondria in HEK^LIPA-Empty^ stimulated for 3h**. Live HEK cells co-expressing LIPA-Empty and mitoGFP were imaged during 5 min.

**Video 3.** **Interaction between LIPA-Empty** **aggregates and mitochondria in HEK^LIPA-Empty^ stimulated for 6h**. Live HEK cells co-expressing LIPA-Empty and mitoGFP were imaged during 5 min.

**Video 4.** **Interaction between LIPA-Empty** **aggregates and mitochondria in HEK^LIPA-Empty^ stimulated for 12h**. Live HEK cells co-expressing LIPA-Empty and mitoGFP were imaged during 5 min.

**Video 5.** **Interaction between LIPA-Empty** **aggregates and mitochondria in HEK^LIPA-Empty^ stimulated for 24h**. Live HEK cells co-expressing LIPA-Empty and mitoGFP were imaged during 5 min. Arrows indicate individual contact between aggregates and mitochondria.

**Video 6.** **Interaction between LIPA-α-syn** **aggregates and mitochondria in unstimulated HEK^LIPA-α-syn^**. Live HEK cells co-expressing LIPA-α-syn and mitoGFP were imaged during 5 min.

**Video 7.** **Interaction between LIPA-α-syn** **aggregates and mitochondria in HEK^LIPA-α-syn^ stimulated for 3h**. Live HEK cells co-expressing LIPA-α-syn and mitoGFP were imaged during 5 min. Arrows indicate individual contact between aggregates and mitochondria.

**Video 8.** **Interaction between LIPA-α-syn** **aggregates and mitochondria in HEK^LIPA-α-syn^ stimulated for 6h**. Live HEK cells co-expressing LIPA-α-syn and mitoGFP were imaged during 5 min. Arrows indicate individual contact between aggregates and mitochondria.

**Video 9.** **Interaction between LIPA-α-syn** **aggregates and mitochondria in HEK^LIPA-α-syn^ stimulated for 12h**. Live HEK cells co-expressing LIPA-α-syn and mitoGFP were imaged during 5 min. Arrows indicate individual contact between aggregates and mitochondria.

**Video 10.** **Interaction between LIPA-α-syn** **aggregates and mitochondria in HEK^LIPA-α-syn^ stimulated for 24h**. Live HEK cells co-expressing LIPA-α-syn and mitoGFP were imaged during 5 min. Arrows indicate individual contact between aggregates and mitochondria.
